# ‘Overcoming and owning challenges’: A qualitative study exploring the manifestation of agency in learners

**DOI:** 10.1111/medu.15631

**Published:** 2025-02-28

**Authors:** Andrew D. Spence, Davina Carr, Anu Kajamaa, Gerard J. Gormley

**Affiliations:** ^1^ Centre for Medical Education Queen's University Belfast Belfast UK; ^2^ General Education Unit, Faculty of Education and Psychology University of Oulu Finland; ^3^ Clinical Skills Education Centre (CSEC), Centre for Medical Education Queen's University Belfast Belfast UK

## Abstract

**Introduction:**

Educators strive to engage learners with their learning. At the heart of this process is empowering individuals to intentionally participate in educational opportunities afforded to them i.e. agentic learning (AL). Significant knowledge gaps remain in how best to promote AL in medical education. Increasingly, simulation is being used to research pedagogical phenomena. In our study, we used simulation as a context to address the following research question: How does individuals' agency manifest in their learning, and what conditions act as enablers for its development?

**Methods:**

Twelve medical students were recruited for this qualitative study. An acute medical simulation‐based exercise was used as a learning context. Following the simulations, semi‐structured interviews were conducted. Recordings of interviews, and simulation pre‐briefs and debriefs were transcribed and thematically analysed using template analysis, drawing upon the Transformative Agency through Double Stimulation framework ‐ to guide our analysis.

**Results:**

We constructed six themes that capture how AL manifests and is promoted. Learners invariably encounter challenges that impede progress in their learning. Such challenges can spur learners to think and act agentially. Feedback provides a mirror for learners to gain self‐knowledge, deepening their commitment to take ownership of their actions and effect change. This process is enhanced by allowing learners to enact and embody new knowledge. Upholding learners' professional credibility and harnessing the social practice of learning – provides conditions for AL to flourish.

**Discussion:**

AL in medical education helps learners shift from *pretending* to *becoming* their future professional selves. By crafting sufficient and facilitated challenges, learners are allowed to ‘hold the tension’ between creatively resolving challenges and upholding their professional credibility. This process is shaped by participation in a social group and can be influenced by the transformation of activities within this group. Embodying changes provides a deep‐seated message that learners can carry to their future.

## Abstract 2

Have you ever considered how we promote learner agency in medical education? In this paper @DrGerryG & colleagues shed new light on the conditions that let learner agency flourish.

## 1 INTRODUCTION

Educators strive to shape the conditions that enable learners to engage with their learning. At the heart of this process is empowering individuals to intentionally participate in the educational opportunities afforded to them. Learners have the potential to drive their learning by focusing on *what* they want to learn and *actively engaging* in the process.^1^, ^2^ Not merely being passive recipients of educational experiences, learner‐centred curricula aim to nurture learners' volition to participate in learning, intentionally think for themselves, and affect change in their professional development.^1^ In the simplest terms, learners are agents (individuals who have the capacity to act), and agency is the manifestation of agents exercising their will to act independently, make choices and enact these choices in the process of their learning.^1^ Despite the concept of agentic learning (AL) gaining significant traction in the broader field of educational research, many consider we lag behind in the medical education research domain.^3^

Agency is a complex and much‐debated concept, with many contending that it lacks a clear and explicit definition capturing its core essence.^3^, ^4^, ^5^ The principles that underpin our understanding of agency draw upon a wide range of disciplines, including psychology, and the social sciences.^6^, ^7^, ^8^, ^9^ In the early 1980’s the seminal work of Mezirov theorised how agency can manifest through emancipatory adult learning.^10^ Over time, many have contributed to constructivist theories of agency in the context of learning.^1^, ^7^, ^8^, ^9^, ^10^, ^11^, ^12^ Prawat theorised that, through a reflective and self‐regulated process, individuals' agency can manifest as they construct knowledge in their learning.^13^ Beyond the individual level of constructing knowledge, Lave and Wenger theorised that the social dimension of learning, as part of a community of knowledge, can enable AL.^12^

In the context of AL in medical education, Watling et al explored how agency manifests in postgraduate medical education.^14^ They highlight that while promoting AL is a challenging “terrain,” there are conditions under which it can be fostered within medical education.^14^ For instance, having “well‐crafted affordances,” such as learner support, opportunities for creativity and safe spaces for discussing risk can create environments conducive to AL.^14^ Their work aligns with established theory, emphasising that agency in learning resides not only within the individual but is also deeply interwoven with the structural and sociocultural influences present in medical environments.^7^, ^8^, ^9^, ^10^, ^11^, ^12^, ^13^, ^14^, ^15^ Despite advancing our understanding of how AL manifests in medical education, they acknowledge that much more research is needed to deepen our understanding of this critical topic ‐ particularly in identifying enabling conditions for AL.^14^

Provocatively described as a “significant omission” in medical education,^3^ we aim to take up this challenge and expand our understanding of AL in medical education. For the purpose of this study, we are influenced by the work of Billett^1^ and Baudrua^2^ in defining AL as an ‘individual's capacity to intentionally think and act for themselves, participate in a learning experience and affect change in their professional development.’

Increasingly, simulation is being used in medical education as a means to research pedagogical phenomena.^16^, ^17^, ^18^, ^19^ In simulation, we recreate realities to facilitate experiential learning.^20^ Unlike the more naturalistic environments of experiential learning, such as the clinical workplace, simulation allows us to design and control environments where individuals can engage and learn.^20^ With a growing evidence base, we are able to narrow the gap between what learners' experience in simulation and in the workplace.^21^, ^22^ The controlled setting of simulation offers researchers a unique window into complex and partially understood phenomena. Moreover, given the degree of control afforded in simulation, we can create valuable opportunities that permit a greater exploration of individuals' implicit and tacit experiences.^23^, ^24^ This includes capturing the immediacy of participants' experiences, thereby reducing reliance on recall and post hoc rationalisation that often occurs long after a learning experience. Therefore, through simulation, we aimed to extend our knowledge of how individuals' agency manifests in learning. Specifically, we posed the following question: How does individuals' agency manifest in their learning, and what conditions act as enablers for its development?

## 2 THEORETICAL ORIENTATION

Numerous paradigms could have been adopted for this study. However, drawing on prior research that shows individuals exercise agency through social participation and by actively constructing knowledge,^7^, ^8^, ^9^, ^10^, ^11^, ^12^ we positioned our work within the constructivist paradigm.

**FIGURE 1:**
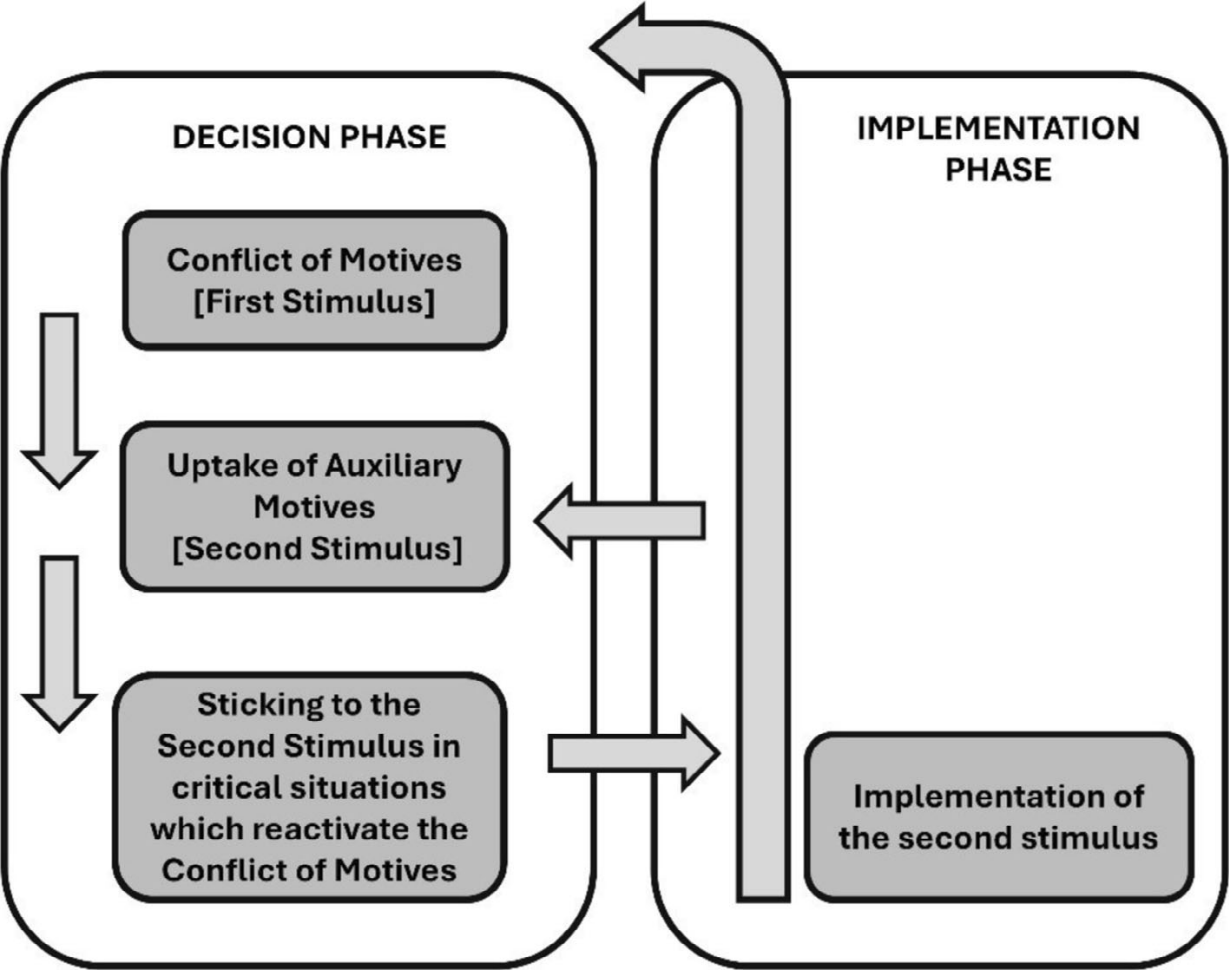
Diagrammatic representation of the transformative agency by double stimulation (TADS) model.^27^

In educational scholarship, Transformative Agency through Double Stimulation (TADS) is increasingly used as an analytical framework to explore how humans agentially transform their learning from individual actions to collective forms of learning.^25^, ^26^, ^27^, ^28^ Sannino's TADS framework elaborates on the concept of double stimulation^29^, ^30^ and provides greater conceptual clarity on how an individual's agency can be transformed through expansive learning (i.e., learning that creates new knowledge for a newly emerging activity) and participation in a collective learning activity (see Figure 1).^27^ They identify two phases in TADS: the ‘Decision phase’ and the ‘Implementation phase’ (See Figure 1).

In the ‘Decision Phase’, we understand that collectives face challenges (i.e., Conflict of Motives) that may hinder their progress (e.g., encountering a challenge in a simulation scenario).^27^ Reflecting on this challenge (i.e., First Stimulus; such as through a simulation debrief) enables individuals to define and understand the issues to be resolved.^27^ Collectively, they critically question current practices and envision new possibilities (i.e., Auxiliary Motives) to overcome these challenges.^27^ Auxiliary Motives, which are either physical or conceptual artefacts, provide a platform for agency and transformation (i.e., Second Stimulus).^27^

In the ‘Implementation phase’, exposing individuals and collectives to conditions that reactivate Conflict of Motives allows them to implement Auxiliary Motives and consolidate new practices.^27^ Providing a collective with a challenge (e.g., a group of students and a facilitator in a simulation) that enables joint analysis and modelling of something new, can act as a driving force for agency and transformation within the group.^27^ Ultimately, the destiny of transformation lies in the hands of the participants, influenced by structural and sociocultural factors.^27^

Given that agency can be considered a collective and social endeavour, and that learners are often challenged to extend just beyond their capabilities, TADS offers a valuable lens for understanding how individual agency manifests in learning.

## 3 METHODS

### 3.1 Study design

We conducted an explorative qualitative interview study.

### 3.2 Setting, recruitment and sampling

The study was conducted at a medium‐sized medical school in the United Kingdom that follows a five‐year Case‐Based Learning model (i.e., a curricular structure that promotes a learner‐centred approach through active student discussions to apply and develop knowledge).^31^ Work‐based learning is the primary form of experiential learning at this medical school, though students also receive simulation‐based education (SBE) opportunities throughout all years, with greater emphasis in the later years. SBE becomes more sophisticated as students' progress. In the junior years, the focus is primarily on technical skills like Cardiopulmonary Resuscitation (CPR), whereas in later years, students engage in more complex simulations, such as team‐based management of critically ill patients.

Convenience sampling was used to recruit participants. We aimed to recruit 9–15 students depending on data sufficiency to address our research question. This sample size range was based on similar study methodologies.^23^, ^32^ Second‐year medical students were chosen for sampling, given their early stage in training and need to develop clinical skills as they transition to more work‐based learning environments. Before this stage, students will have had exposure to SBE, including CPR and Automated External Defibrillator skills. An invitation email was sent to all second‐year students, and participants were selected on a first‐come, first‐served basis.

### 3.3 Description of the SBE exercise

For this study, an SBE exercise was designed focusing on the assessment and management of a patient experiencing cardiac arrest (see Appendix S1 for full details). Participants engaged in a simulated scenario with a ‘patient’ (a full‐body simulation manikin) who initially complained of chest pain and later suffered a cardiac arrest. The simulation took place in a simulation suite, with participants working in teams of three. The facilitated scenario included a pre‐brief and debrief. During the debrief, participants could implement new knowledge by re‐running elements of the simulation. The facilitator aimed to create a psychologically safe environment through comprehensive pre‐brief and debrief sessions, promoting mutual respect and encouraging participants to express their views without fear of retribution.^33^

### 3.4 Data collection

All pre‐brief and debrief discussions were recorded using a digital Dictaphone. After the simulation, individual semi‐structured interviews were conducted by GG, AS and DC. All interviewers met to ensure consistency in their approach by gaining a shared understanding and use of the interview guide (Appendix S2) and methods of interviewing.^34^ After the interviews, the interviewers collectively debriefed to maintain consistency in their approach.

The interview guide (Appendix S2) was developed based on concepts of how individuals learn in SBE, focusing on participants' experiences of the simulation and the factors that motivated their learning (including instances of First Stimulus, Conflict of Motives, Secondary Stimulus, Auxiliary Motives and experiences of implementing Auxiliary Motives).^27^ Interviewers were encouraged to be curious and create a dialogic space to support emerging discussions. We did not define the term AL for participants to avoid priming them with pre‐conceived ideas.

All pre‐briefs, debriefs and interviews were audio‐recorded and transcribed verbatim. Transcripts were checked for accuracy, and pseudonyms were assigned to participants. The simulations were conducted on different dates, allowing data analysis to occur concurrently with data collection.

### 3.5 Data analysis

A Template Analysis^35^ approach was used for data analysis, with NVivo® qualitative software (Version 12.7.0) aiding in our organisation and categorisation of data.^36^ Our analytical procedure was initially (inductively) data‐driven. Two contrasting interviews were first selected to ensure a range of perspectives. Two researchers (GG and AS) independently reviewed these transcripts, discussed any disagreements in their coding until there was agreement and devised an initial template. Then abductively (i.e. our analytic approach involved repeated iterations between theory and data^37^, ^38^) we used elements of the TADs framework (i.e., First Stimulus, Conflict of Motives, Secondary Stimulus, Auxiliary Motives and implementation, along with enabling conditions),^27^ to draw upon in our analysis and development of the template. Through regular meetings, the template was iteratively developed and modified based on the application of all transcripts. Researchers met frequently to share their analysis and conduct comparative analysis across all transcripts until achieving theoretical sufficiency (i.e., reaching a sufficient level of derived meaning to address the research question), ensuring consensus that the final themes provided a rich and thick description.^39^

### 3.6 Reflexivity

The research team maintained continual reflexivity throughout the study, engaging in open discussions about their assumptions and potential influences on the analysis.^40^ They recognised that their differing perspectives could enrich the research. GG is a clinical academic (General Practitioner) and professor of simulation; AS is a clinical academic (Gastroenterology) with an interest in simulation; and DC is a practicing General Practitioner and clinical teaching fellow with an interest in simulation. Given their shared interest in simulation, AK provided a critical counterbalance. AK is an educational scholar with no background in simulation and did not work at the institution where the other authors were based. AK offered an external, critical perspective on the analysis and approach to the study. Importantly, none of the research team had direct teaching relationships with the study participants at the time of the study.

## 4 RESULTS

We conducted four simulations (1–4) and interviewed 12 participants (average duration of semi‐structured interviews: 27.05 minutes). Five of the subjects identified as being male and seven as female. Table 1 conveys the main parameters of the simulations.

**TABLE 1:** Main parameters of the simulation activities conducted in this study.

Simulation session	Participants involved (pseudonyms)	Duration of pre‐brief (mins:secs)	Duration of simulation (mins:secs)	*Duration of debriefing process (mins:secs)
1	Agata, Muhammad and Stephen	5.31	21.45	42.42
2	James, Archie and Grainne	3.13	20.23	29.35
3	Janet, Martina and Juno	3.15	22.39	24.24
4	Nick, Melissa and Sam	4.20	24.43	35.29

* Includes time spent in the debrief including time to apply new knowledge within a further segment of a simulation.

**FIGURE 2:**
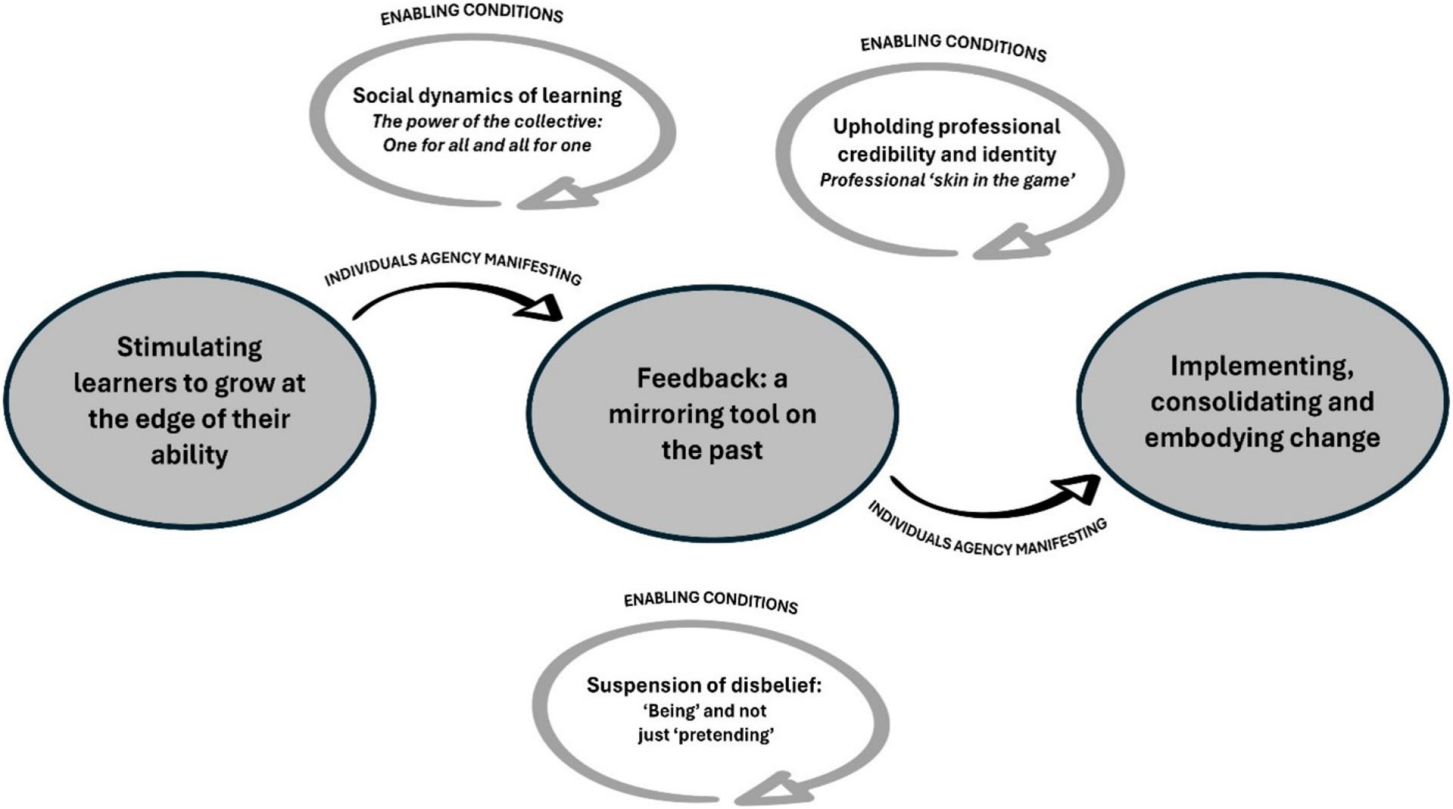
Map of themes developed in this study. [Color figure can be viewed at wileyonlinelibrary.com]

Our analysis identified six interrelated themes (see Figure 2). These themes help to understand how individual agency manifested in learning and the conditions that influenced its promotion. Below is a detailed description of the themes, along with participants' quotes, to convey the findings.

### 4.1 Stimulating learners to grow at the edge of their ability

By intention in the design of the simulation (based on the participants' stage of their studies) we envisaged that they would encounter challenges (i.e. Conflicts of Motives)^27^ that would impede their progress in the simulation. Whilst such problematics could impede their immediate progress, these stimuli had the ability to spur learners to agentially think, and act, for themselves. As the simulation unfolded, participants engaged with the scenario, driven by the desire to achieve the outcomes of the learning exercise (e.g. learning how to provide better patient care). Some challenges were perceived as trivial and within reach of participants' capabilities, whereas others extended them. One participant narrated about their group's challenge in operating the hospital bed:

‘… I guess one of the main problems we had, well as a group ‐ the positioning of the patient … . We didn’t have the bed maybe as flat the way we should have. We weren’t as familiar with the equipment … . that can be quite frustrating especially in a situation when you feel like you’re under time pressure.’ (Muhammad; Debrief)

Participants, collectively, would be driven to consider what was impeding their progress and envisage new possibilities and ideas (i.e. Auxiliary Motives)^27^ to overcome these challenges (i.e. Conflicts of Motives).^27^ Participants voiced that having to ‘think on their feet’, which mediated engagement in their learning. Being able to create new possibilities, turn them into productive instruments (i.e. virtual or physical) and intentionally wanting to act on these (i.e. Second Stimulus),^27^ was a motivating force to overcome the challenges. As one participant described the need for leadership in the group:

‘But then when the whole situation deteriorated, and we all needed to get involved I don’t really think we had a leader. We all were just trying to think of something to do and then do it, and then everyone else was working around you. We had to overcome this’ (Nick; Interview)

Being extended beyond their capabilities gave participants a sense of responsibility more than what they were normally used to; driving their willingness to take ownership of their actions as they learned at the edge of their abilities.

‘Well, I think just in your head like you know it's really important that you have to just keep going. Like do everything you can for the patient and you kind of have to compress those feelings, keep them under control until the simulation is finished’ (Martina; Interview)

### 4.2 Feedback: a mirroring tool on the past

Participants voiced the importance of feedback (in this instance the debrief) on their engagement in learning. Through these facilitated discussions, participants described how they gained self‐knowledge and awareness about their abilities (i.e. First Stimulus).^27^ In this process, facilitators would not only validate individuals' experiences but identify blind spots about their performance (i.e. aspects of their actions in the simulation that they were not able to recall). As one participant explained:

‘… .I feel like once you start talking [In the debrief] … . you start realising, oh my god, did I actually do that? And did it happen? You start realising what actually happened and what was going on throughout’ (Grainne; Interview)

Such a process of recalling their performance provided a *mirror* of their capabilities and inabilities. In so doing, it enhanced participants' self‐knowledge and ability to critically reflect on their performance. Participants explained that they gained a greater sense of ownership of their actions, which drove their intrinsic desire to make a change in their future professional self. As described by one participant:

‘I think sometimes you need to … . take a step back and have a talk through things you can actually see yourself what you need to do and where you can improve. It's quite a good feeling when you can sort of identify what you need to do differently. Especially when you feel like you’re doing it yourself. It makes you feel quite positive about doing those kind of things in the future.’ (Stephen; Interview)

Invariably, participants would consider the challenges (i.e. Conflicts of Motives)^27^ that they encountered within the educational exercise. Not only those that they were able to overcome, but also those challenges that they failed to resolve and often explored in the debrief (i.e. First Stimulus).^27^ Whilst recalling what went well was often positively received by participants, exploring what could have been better often commanded much more of their attention. Helped by a supportive relationship with the facilitator, participants could envisage new ways to overcome these challenges (Auxiliary Motives)^27^ if they encountered them again. One participant explained:

‘… once you see the better way to do it, it's a lightbulb moment. Oh, that railing pops down or that bit of the bed comes off, it's like oh okay I didn’t know that before. I know it now. I didn’t feel like stupid or, you know, I wasn't slapping myself on the head for having known that. It was like okay that's how you do that. (Stephen; Interview)

### 4.3 Implementing, consolidating and embodying change

Universally, participants expressed that the experience of implementing their newly created knowledge (Second Stimulus)^27^ was a motivating force to affect change in their future professional selves. As expressed by one participant:

‘I'm definitely going to remember much more if I was just told that information. Like trying to hold onto it and then walked off, I feel like probably would have just gone out of my head and then I would have come on the next one probably a bit better but not knowing everything. Whereas I feel actually running through and actually getting hands on, doing the process is so useful. (Nick; Debrief)

Following the simulation, participants would often co‐create new knowledge (Auxiliary Motives)^27^ of how to overcome challenges and conflicts (i.e. Conflict of Motives)^27^ that they encountered. Rather than just leaving this as a cognitive exercise in the debrief, being offered the physical opportunity to encounter the original challenge again provided a pivotal moment in AL. Reactivating such Conflicts of Motivates and physically implementing their auxiliary motives often evoked an influential embodied experience.

‘The muscle memory. Everything just, it will stick in my head because I'm actually physically doing it.’ (Juno; Interview)

Enacting their new knowledge made it personal to them and helped consolidate their learning. Such an experience mediated their sense of self‐fulfilment which could be taken forward in their future practice. Moreover, being able to overcome this challenge, demonstrated to themselves and others – they had transformed. Interestingly, the implementation of their new knowledge often evoked positive emotions, such as pride, and enhanced self‐knowledge and professional identity. In so doing, complemented their desire to bring this change to their future self.

‘Because I think the first time you do it, you sort of leave it and you're like kind of negative … .I should have done this, or I could have done that. Whereas the second time you are sort of getting the sense of accomplishment. Like oh, I did that well or I'm actually more confident than if I run the situation again in a real‐life scenario.’ (Janet; Interview)

### 4.4 ‘Being’ and not just ‘pretending’

In terms of conditions that were conducive to AL, universally, participants articulated that being able to buy‐into the educational experience was an important condition to engage in their learning. Shifting their sense that they were *being* present with a more real‐to‐life experience linked to their professional context, rather than just *pretending*, provided an important basis to intentionally drive forward their learning. As one participant described, being immersed in the activity enabled them to suspend disbelief and provided an impetus to engage and care for the ‘patient’:

‘… I think today the whole set up was really quite immersive with the ward; how well everything was set up … .it felt very life like and really helped. It made me want to do even more [for the patient]’ (Muhammad; Interview)

Across our dataset, participants provided insights into the many enablers that helped them to engage in the activity. Typical of SBE environments, the setting aims to recreate a clinical context. Artefacts such as hospital curtains, trolleys, beds and clinical equipment all evoked a sense of place and meaning for participants. Though participants knew this was not real life, having an openness to temporarily suspend disbelief mediated engagement. The context of having a high‐acuity event appeared to mediate participants' suspension of disbelief and deepen engagement. As explained by one participant:

‘… .it definitely felt a bit of adrenaline and kind of rushing around trying to grab everything, grab the defibrillator. Like I felt the pressure to work quickly because it felt like a serious scenario.’ (Nick; Interview)

### 4.5 Professional ‘skin in the game’

Upholding professional credibility in the educational activity was an enabler for AL to manifest. By ‘performing’ in the simulation, they externalised their professional capabilities to others. In so doing, participants expressed that this often drew attention to self‐reflection, self‐knowledge and professional identity. Many articulated this motivated their engagement in the simulation:

‘I think some of my techniques there I was slightly embarrassed about especially with the fact that there is other medical students there ‐ you wanted to do the right thing … . and I'm sure that will be the same in the hospital when there's staff there. But I think I came away from it and I wanted to do better the next time for the patient’ (Archie; Debrief)

During the educational activity, participants often felt under the gaze of others, namely fellow students and facilitators. This created a sense of scrutiny. Even if they personally knew others in the simulation, they often felt judged. As articulated by one participant:

‘… I felt quite nervous going into it. But knowing especially not even that there's doctors around but there are my colleagues and the people I learned with every day, the other medical students. I didn't want to show it because I thought what if they know it and I don't? So, it was just trying to remain confident and remain calm.’ (Sam; Interview)

Having a sense of scrutiny, heightened self‐awareness on their professional standing. Whilst this had the potential to help develop their professional identity, more often participants experienced this as a potential threat. Conversely, this potential threat to their professional identity appeared to be a motivating force to engage in their learning. As explained by one participant:

‘I don't want to say useless but, in a way, you just feel ashamed of yourself. Because you want to be prepared. You want to be good, you want to be a good doctor at the end of it’ (Grainne; Interview)

Despite this sense of judgement, there was an opportunity for participants to counterbalance such perceptions to help them learn. This was particularly evident in the simulation debrief. Given that this was a collective activity, it could help foster their ability to shift their belief that the educational activity would help them, and others, be better doctors:

‘Obviously it's never nice to not know what you're doing in front of your peers, but I definitely didn't feel as bad as I might have done or as I could have done, or have at other times. I felt quite comfortable today. I felt like everyone was in the same boat and those guys were in the same position as me, so I didn't feel too bad.’ (Muhammad; Interview)

From this position, participants gained greater ownership of their actions in the educational activity. Moving from the notion of *being a medical student* with limited responsibility ‐ to experiencing a degree of responsibility in *becoming a future doctor*.

### 4.6 The power of the collective: *One for all and all for one*

The social dynamics within their learning influenced how participants intentionally engaged in their learning. The collaborative nature of simulations, as experienced by participants in this study, involved interaction with their peers and facilitators. Participants described how these social dynamics played a crucial role in facilitating AL:

‘You go in … kind of knowing each other … ..to at the end … .you trust each other … . the communication and the teamwork was there. I did say to them, “Oh, can you please do the CPR and can you please call an ambulance?” And I knew in a way they were going to get it done … Whereas at the beginning I didn't really know them that well and it kind of built up our relationship and trust in progressing the simulation.’ (Grainne; Interview)

As described in an earlier theme, participants often felt under the gaze of others. This drove participants to focus on their performance and strive to portray their professional creditability amongst their colleagues. With time, participants explained they gained a greater shared purpose. They began to forge a bond with their colleagues and support each other for the common good of providing ‘patient’ care. This marked a shift from being an *individualistic* to a more *collective* endeavour. As expressed by one participant:

‘… you kind of bond together when you're doing something like this … .there's camaraderie and because you've all gone through something which is relatively intense although it is simulated … it's still at the time it is quite immersive it does feel pretty intense. So, you get a bond with each other and working together like that is quite nice … . you feel good talking to each other about it afterwards.’ (Muhammad; Interview)

In making such supportive connections, there was a greater sense of being able to share their struggles and being open to suggestions of how to advance with the simulation. Importantly, participants expressed how challenges became a shared responsibility.

‘And I was also thinking of the team itself and how we can utilise the team and that it wasn't just my responsibility it was the responsibility of the team around me.’ (Sam; Debrief)

This shift to a more collective perspective provided the space for participants to voice their challenges (Conflicts of Motives),^27^ creatively think of solutions (Auxiliary Motives)^27^ and engage in the exercise as if it were real life.

## 5 DISCUSSION

Despite its complexity, our research provides new insights into how individuals' agency manifests within the context of learning in medical education. Rather than being passive recipients of educational efforts, learners can, under certain conditions, be empowered to develop agency. Through the theoretical lens of TADS, we identified several conditions that enable AL. First and foremost, our findings support the notion that AL is a co‐constructed process that requires effort; it does not occur by chance. As Watling et al asserts, fostering agency requires work.^14^ However, this effort is worthwhile, as promoting agency can have a positive impact on learning (Billet).^1^, ^41^

A key finding of our study is that challenges encountered in learning can serve as a springboard for AL. Learners often face conflicts that hinder their progress in a learning activity. However, as our research demonstrates, such conflicts can create pivotal *moments* for fostering AL.^27^ Rather than hindering agency, these conflicts have the potential to capture an individual's attention and motivate them to resolve such challenges. This finding aligns with Sannino's work on TADS in that can enable agency^27^ and asserted by Virkkunen that “breaking away from the given frame of action and taking the initiative to transform it”.^42^

Crucially, our research highlights the importance of adequate support when learners encounter challenges. Feeling supported can empower learners to take ownership of their learning, envision strategies to overcome conflicts and take measured risks in the process. This finding aligns with Prawat's work, which emphasises that individuals exercise agency through a supportive and reflective problem‐solving process whereas constructing knowledge.^13^ Furthermore, our research suggests that when learners feel supported during such challenges, they are better able to agentially envisage artefacts that can serve as Auxiliary Motives to overcome Conflicts of Motives in these difficult situations.^27^

Our research identified numerous instances that highlighted the importance of the socio‐relational aspects of AL. Agency in learning does not reside solely within the individual but is interdependent on the social context of their learning.^7^, ^8^, ^9^, ^10^, ^11^, ^12^, ^13^, ^14^, ^15^, ^43^ A person's agency can be empowered through social groupings and processes. Whether garnering support from peers or educators, establishing the socio‐norms that permit risk‐taking, or accessing appropriate conceptual artefacts provided by others, enacting agency is inherently a social phenomenon.^7^, ^8^, ^9^, ^10^, ^11^, ^12^, ^13^, ^14^, ^15^, ^43^ These findings align with the work of Lave and Wenger, who assert that agency can be enabled through active participation within a knowledge community.^12^ Furthermore, they resonate with Freire's (1973) perspective that social empowerment can enable agency.^44^

A further key finding of our research is that individuals' agency is closely connected to their sense of autonomy and self‐fulfilment. By fostering a sense of ownership in their learning, individuals are more likely to manifest intentional actions in their educational activities. This finding aligns with Giddens' work, which emphasises that agency is evident when individuals act as agents of change, taking creative initiatives to improve existing practices.^8^, ^45^ The relational aspects of AL are deeply interwoven with learners' belief that their efforts contribute to the development of their professional identity.^43^ They are not merely *pretending*; but striving to *become* a more capable, future professional self. Moreover, individuals need to feel that their learning is making a meaningful contribution to their professional development. When learners believe they are in control of their learning trajectory, they experience a strong sense of ownership and actively shape their professional identity. These findings resonate with TADS, where ownership and transformation of the activity lie in the participants' hands.^42^

A particularly intriguing finding from our research is individuals' embodied sense of learning. Developing an embodied relationship with their learning can enable individuals to experience greater self‐fulfilment and deeper buy‐in to their learning. This aligns with Kelly's work,^46^ which emphasises the importance of such embodied connections in the learning process.^46^ Exposing learners to embody the conditions that reactivate Conflict of Motives allows them to gain control, implement envisaged Auxiliary Motives and transform a problematic situation.^27^ As a result, both the problematic situation and the learner are transformed.

### 5.1 Limitations

Despite the novel findings of our research, they must be considered within the limitations of this study. Simulation was used as a setting to investigate how individuals' agency manifested in their learning. Given the controlled nature of simulation, this approach provided a unique window to access learners' in‐depth and tacit experiences. However, the findings of this study may not be readily transferable to other contexts, including other forms of learning such as work‐based education. For example, we recruited medical students as learners in this study. Although this permitted insights into how individuals' agency emerged at this stage of their training, it may limit the transferability to other stages of learning (e.g., postgraduate continual professional development). The simulation scenario used in this study pertained to an acute clinical situation; individuals' agency may manifest differently in other situations (e.g., learning about chronic disease). Though the setting in which we conducted our study was a typical UK medical school, findings may not be readily transferable to other medical schools and institutions. In our study, learners physically applied new knowledge gained during debriefing, an approach not universally practiced in SBE. Nonetheless, this research elaborates our understanding in this under‐researched area, and the novel use of a theoretical framework (i.e., TADS) goes some way in aiding the transferability of our findings.^27^ Our research also stimulates future lines of inquiry, including how individuals' agency in learning is influenced by their stage of studies, the context of their learning (e.g. in work‐based learning), interprofessional learning and the nature of the subject matter they are learning. Moreover, it would be worthy of future research to explore the impact of power, gender and other characteristics on individuals' agency in learning and the role of a facilitator to promote AL.

### 5.2 Implications for educational practice and future research

Our research findings offer several implications for educational practice. As educators, we have the potential to modify the design and conditions of learning for our students. Therefore, through educator training, it is essential to emphasise where to focus our efforts in promoting AL. Not only by raising the profile of AL in educator training but also by promoting the conditions that enable AL and endorsing such practices.^47^

Firstly, where possible, we should focus on the social dimension and dynamics of learning. Allowing students to learn together can foster a collective sense of ownership and engagement in their learning. Secondly, providing learners with sufficient challenges, but within their capabilities, can activate their agency to overcome these challenges in the pursuit of their learning. Therefore, through educational design, we should strive to craft sufficient challenges for our learners. Feedback is critical in this process, helping learners gain self‐knowledge and reinforcing their sense of ownership of their learning and actions – i.e., they are not merely ‘pretending’ but enacting their future professional selves. Thirdly, by allowing learners a sense of ownership in their actions and through supportive educator relationships, we can enable them to engage in their learning and develop their sense of professional credibility and identity.

Finally, allowing learners to implement their newly acquired knowledge can act as a powerful motivator in their learning. Where possible, allowing learners to consolidate their learning through practical application can enable an embodied sense of taking such new knowledge to their future professional self.

## 6 CONCLUSIONS

The importance of social processes in promoting AL should not be underestimated. Individuals' agency can be empowered through social dynamics and collaborative relationships within a group. By presenting sufficient challenges, educators can enable AL. Supportive educator relationships empower learners to actively envision and develop new strategies to address these challenges, enhancing their sense of control and deepening engagement. Learners' professional identity formation plays a crucial role in this process and is closely tied to AL. Understanding that learning is not merely a process of *pretending* but a key part of *becoming* their professional self can drive AL. Allowing learners to embody new knowledge, rather than just intellectually engaging with it, strengthens their sense of control as they actively shape their future professional self.

## AUTHOR CONTRIBUTIONS

All authors contributed to the conceptualisation of this study. Gerard Gormley (GG), Andrew Spence (AS) and Davina Carr (DC) conducted the study and collected data. All authors participated in data analysis. Manuscript writing and editing were led by Gerard Gormley and Andrew Spence, with input from the other authors. All authors approved the submitted manuscript.

## CONFLICT OF INTEREST STATEMENT

The authors report no conflict of interest. The authors alone are responsible for the content and writing of this article.

## ETHICS STATEMENT

Ethical approval was provided by the Faculty of Medicine, Health and Life Sciences Research Ethics Committee at QUB. Informed written consent was obtained from all participants.

## Supporting information

Appendix 1: Details of the SBE learning activity.

Appendix 2: Interview guide.

## Data Availability

The data that support the findings of this study are available on request from the corresponding author. The data are not publicly available due to privacy or ethical restrictions.
