# Supplementary material for: ‘Overcoming and owning challenges’: A qualitative study exploring the manifestation of agency in learners
Source: Med Educ. 2025 Feb 28;59(9):972–82. doi: 10.1111/medu.15631 (PMC12355631; doi:10.1111/medu.15631)
Supplement: Supplementary file 1 — Appendix 1: Details of the SBE learning activity. [file MEDU-59-972-s002.docx]

**Appendix 1: Details of the SBE learning activity**

The simulation was specifically designed for the level of knowledge and abilities of medical students at this stage of their training. In summary, the simulation focused on the assessment of a ‘patient’ in a hospital ward that was experiencing chest pain. Using a full body humanoid manikin, participants were asked to assess the ‘patient’ (Figure 3). During the course of this assessment, the patient went into a cardiac arrest. Participants were asked to respond appropriately to this emergency. Ideally, students would commence CPR, summon the cardiac arrest team and apply an AED to administer an electric shock. Prior to this point in their training, students will have had some simulation experience of these skills, but this exercise was intended to extend their abilities by applying all of these skills in a simulated hospital setting and overcome some of the new contextual factors to them. Groups of three students participated in the simulation at one time, to promote team-based and human factor skills. The simulation was conducted in a simulation suite with a humanoid manikin and clinical equipment. The research team were able to observe the simulation via a one-way mirrored screen.

In accordance with good simulation practice, participants received a simulation pre-brief prior to engaging with the simulation. A debrief was then conducted using the PEARLS model [18]. During the debrief, and in keeping with PEARLS, participants were asked to articulate new ways of practice that arose as a result of this experience. For the purposes of this study, participants were offered to repeat elements of the simulation to implement their envisaged new ways of practice, and a brief discussion about this experience. The simulations were facilitated by a simulationist who strived to ensure psychological safety throughout the simulation and provide directional flow to the simulation.


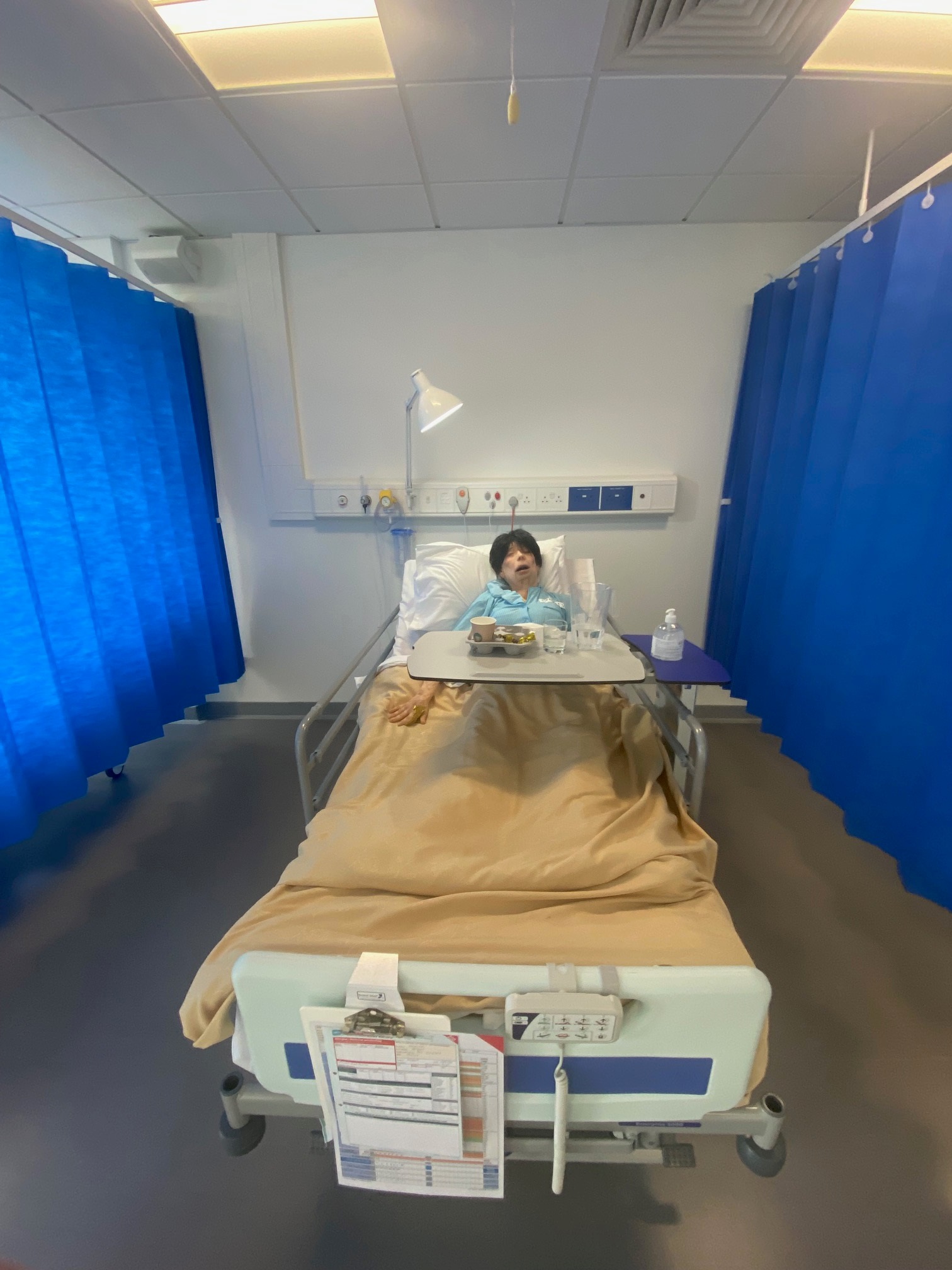


**Figure 3. Image of simulation environment used for the purposes of this study**
