# Supplementary material for: ‘Overcoming and owning challenges’: A qualitative study exploring the manifestation of agency in learners
Source: Med Educ. 2025 Feb 28;59(9):972–82. doi: 10.1111/medu.15631 (PMC12355631; doi:10.1111/medu.15631)
Supplement: Supplementary file 2 — Appendix 2: Interview guide. [file MEDU-59-972-s001.docx]

**Appendix 2 Interview guide**

Hello, my name is [interviewer name], and I am an academic in the Centre for Medical Education. Thank you for taking the time to participate in this study.

The purpose of this interview is to explore your experience of the simulation exercise that you just conducted. I predict that this interview will last approx. 30-60 mins. During this interview you will be asked questions to stimulate a discussion about your experiences in the simulation exercise that you just took part in. There are no right or wrong answers to any of the questions and if there are any questions you do not wish to answer, please say so and we will move on. The purpose is to facilitate a conversation and allow you to express your experiences. I hope you will feel comfortable being honest about your experiences with me.

This session will be recorded so that I can adequately capture your ideas and conversation. However, your comments from the interview will remain confidential and you name will not be attached to any comments you make. The interview will typically last 45 minutes but may range from 30-60 minutes. You are free to stop the interview or withdraw before we begin. Do you have any questions before we begin?

To begin with could you please share with me your experiences about the simulation exercise that you just conducted? Can we start with the Beginning (briefing), First simulation, Debrief, Second simulation and Final debrief?

Please do share you experience about the simulation.

How did these experiences make you feel?

Did you have any challenges in the simulation? How did you overcome them? What was this experience like?

How did you feel about this discussion with others of how you got on in the simulation?

How do you feel about generating new approaches following the simulation?

When you finished the debrief – do you feel you could change your practice? What made you think like that?

In what ways did you think the second simulation make a change for you – if any?

Do you think these experiences will influence your future practice? If so – in what way?

What do you think were the influences on your experiences?

Do you have any other experiences you would like to share?

Thank you for your time and for sharing your experience with me. Do you have any final questions or comments?
